# Integration of the work-related online aftercare intervention *‘GSA-online plus’* (healthy and without stress at the workplace) into clinical practice: study protocol for an implementation study

**DOI:** 10.1186/s12913-018-2995-z

**Published:** 2018-05-02

**Authors:** Rüdiger Zwerenz, Katja Böhme, Astrid Wirth, Nicole Labitzke, Sergei Pachtchenko, Manfred E. Beutel

**Affiliations:** 1 Department of Psychosomatic Medicine and Psychotherapy, University Medical Centre of the Johannes Gutenberg-University, Untere Zahlbacher Str. 8, 55131 Mainz, Germany; 2 German Reading Foundation, Mainz, RP Germany; 3 Media Centre of the Johannes Gutenberg-University, Mainz, RP Germany; 4 Knowledge Media Institute of the University of Koblenz-Landau, Koblenz, RP Germany; 5 Department of Clinical Psychology, Psychotherapy & experimental Psychopathology, Johannes Gutenberg University, Mainz, Germany

**Keywords:** Internet-based intervention, Rehabilitation aftercare, Psychological online support, Return to work, Work stress, Implementation study

## Abstract

**Background:**

In a previous RCT we established the efficacy of the psychodynamic online aftercare programme *‘GSA-Online’* (‘Health Training Stress Management at the Workplace’) for rehabilitants with work-related stress facing return to work after long-term sickness absence. The purpose of this trial is to implement it into routine care.

**Methods/design:**

The study is performed in rehabilitation clinics with patients of different medical indications (psychosomatic, orthopedic and cardiological diseases). Rehabilitants get access to the study platform during inpatient medical rehabilitation. *‘GSA-Online plus’* integrates exploratory and motivational videos on the web application to familiarize potential participants and motivate them to follow through with it. In the 12-week writing intervention, patients write weekly online diary entries, answered by anonymous online therapists within 24 h. Primary outcome measures are the recommendation rate of *‘GSA-Online plus’* and participation rates of the rehabilitants. As secondary outcomes, psychological symptoms, overall satisfaction, helpfulness of the therapeutic feedback and utilization of *‘GSA-Online plus’* will be analysed exploratory along with the course of weekly ratings of well-being and work ability.

**Discussion:**

Meanwhile many clinical trials and meta-analysis prove that internet-based interventions are effective. This study will add insights on the dissemination and implementation of efficacious, evidence-based online treatments into medical practice. We expect a successful implementation of *‘GSA-Online plus’* in the clinical routine of the rehabilitation clinics. The focus of evaluation is on acceptance of the programme, both by the physicians in charge and the patients. In the future *‘GSA-Online plus’* could be implemented as a routine aftercare programme for rehabilitation inpatients with occupational stress.

**Trial registration:**

The trial was retrospectively registered on 6th January 2017 at ClinicalTrials.gov (Trial Registration number: ClinicalTrials Gov ID NCT03019718).

## Background

In Germany, inpatient medical rehabilitation has been implemented for patients with chronic mental and somatic complaints in order to restore and maintain work ability [1]. Approximately one third of the German population has reported significant work-related stress [2]. As we found previously, inpatients of psychosomatic rehabilitation clinics reported not only higher work-related stress, but also fewer coping resources when compared to the general German population [3]. Therefore, various work-related interventions have been adopted during inpatient medical rehabilitation treatment in order to deal with work-related stress [4]. In a previous clinical trial we could show that vocational training during inpatient psychosomatic rehabilitation improved return to work in the long run [5]. A recent meta-analysis of randomised trials for work-related medical rehabilitation interventions in patients with musculoskeletal disorders showed better return to work outcomes compared to usual medical rehabilitation [6].

Applying psychological and practical strategies acquired in rehabilitation into daily work remains a critical obstacle for many patients with chronic mental or physical disorders, particularly if they have already had long periods of sick leaves. Aftercare interventions have therefore been implemented in order to support return to work and participation in social life [7, 8]. However, few patients take part in outpatient treatments following inpatient rehabilitation because of incompatibility with their duties at work or within their families, or poor access to the outpatient rehabilitation facility [9, 10].

Internet-based interventions appear to be promising, as the great majority of the German population (84% in 2016) is online, and the internet is increasingly used for health- related issues [11, 12]. Under the heading of occupational e-mental health, internet-based interventions have been applied to deliver education, health risk assessment, work-place health promotion, preventive interventions, treatment, relapse prevention, and return-to-work assistance [13]. But online interventions focusing directly on workplace reintegration after inpatient rehabilitation are still missing. Furthermore, acceptance and uptake of online interventions is generally still limited [14] and dropout rates for psychological online interventions are often elevated [15, 16].

In order to promote successful vocational reintegration after inpatient medical rehabilitation we devised a transdiagnostic psychodynamic online aftercare programme for patients with chronic diseases (psychosomatic, cardiological, orthopaedic) [17]. This is one of the few examples of online interventions, based on a psychodynamic model [18–21]. As we could show recently, hassles with colleagues or superiors play a major role for work-related stress, fatigue and depression [22]. Another study, analysing expectations of patients toward case management after psychosomatic rehabilitation, found that most patients required support concerning conflicts at the workplace [23]. Therefore we chose a psychodynamic concept focusing on interpersonal conflicts at the workplace in our prior study [17]. In weekly writing tasks, participants were instructed to describe interpersonal situations at the workplace according to their wish, reactions of the other and their own reactions. Following the concept of the ‘Core Conflict Relationship Theme (CCRT)’ [24], the online therapist identified maladaptive relationship patterns and provided written feedback. In a randomised controlled trial (RCT) with a total of 664 participants (vs. an information only control group), we could show that the online intervention was reasonably accepted (78% of log-ins, 66% writing blogs).The majority of participants did not use the online intervention continuously, but rather six times (M = 6.00; SD = 4.21) during the possible 12 weeks [25]. Overall, psychodynamic online aftercare was effective to enhance subjective prognosis of future employment and improved psychological complaints (anxiety, depression, somatisation) as well as quality of life across a variety of chronic physical and psychological conditions, albeit with small effect sizes [25]. While most previous trials recruited over the internet, one of the strengths of our previous RCT was that we recruited patients from the rehabilitation clinics. As usual in RCTs, enrolment of participants was done by research staff based on screening criteria assessed by questionnaire. Recruitment also included a brief group intervention in each clinic by trained clinicians in order to familiarize participants with the programme and its rationale and to motivate them for participation.

## Methods/design

### Study design

**Fig. 1:**
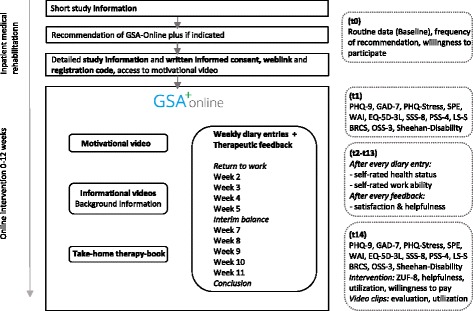
Overview of the course of the trial

Figure 1 gives an overview of the course of the trial.

The current study is a single group assignment with an anticipated sample size of *N* = 212 participants. We perform a combined cross-sectional and longitudinal assessment. Based on clinic routine documentation all patients admitted during the recruitment period are documented. All patients who obtain an aftercare recommendation of *‘GSA-Online plus’* and give written consent to take part in the study are followed longitudinally.

### Participants and recruitment

Recruitment is performed in three rehabilitation clinics specialized for psychosomatic, oncological, orthopaedic and rheumatic as well as cardiological diseases, which had not been involved in the previous trial. Physicians in charge received written information material about *‘GSA-online plus’* and were instructed about the inclusion criteria before recruitment started, however no formal screening of patients is involved. Patients are eligible if they a) are employed and return to their workplace within 4 weeks after inpatient medical rehabilitation, b) are able to write in German language, c) are between 18 and 59 years old and d) have a private internet access. The presence of comorbid mental disorders is not an exclusion criterion. A severe physical or mental disorder requiring an intensive treatment after rehabilitation precludes recommendation of online aftercare. Physicians are suggested to recommend the programme if indicated, according to their judgement (e.g. anticipated problems in vocational reintegration). This decision does not affect referrals for other aftercare programmes. In any case the physician responsible documents referrals for *‘GSA-Online plus’* or other aftercare recommendations and the agreement or refusal of the patient.

Patients also get a brief study information about *‘GSA-Online plus’* upon intake to rehabilitation by the medical director of the rehabilitation clinic. If they either report occupational stress or a subjective need for occupational treatment during inpatient medical rehabilitation, they may inform the clinical staff (e. g. physician, psychologist, social worker) to judge if participating at *‘GSA-Online plus’* is indicated. If patients are deemed suitable by the clinician and are interested in participating, they receive detailed verbal and written information about the study participation by a clinical employee. They also obtain access to the internet platform. Before registration all participants watch a video clip to obtain information about content and procedure of *‘GSA-Online plus’*. During the registration process all participants are asked to choose a nickname and a password and have to enter their email-address. The email-address is essential so that the participants will be informed by automated emails about study-related information, therapeutic tasks and questionnaires to be filled out. Subsequently, they log in with their nickname and a password. Then they may use information modules (video clips and written text) which aim at preparation and motivation of participants. After discharge they are able to participate fully in the 12-week programme.

### Intervention

#### Inpatient treatment

Depending on the specific chronic disease or impairment, inpatient medical rehabilitation covers diagnostic and therapeutic interventions, health education, physical training, and psychological support as indicated. At treatment termination, the responsible physician provides an assessment of work ability and of measures to improve or maintain work ability in the long run, which may include face to face aftercare interventions.

### Online intervention

The web application *‘GSA-Online plus‘* is based on the previous *‘GSA-Online‘*, which had required introduction into the programme by a clinician-led psychoeducational group [17]. *‘GSA Online plus‘ *was re-implemented from the ground up using modern web development technologies and responsive web design. It offers additional explanatory and motivational videos on the study web application to familiarize potential participants with the internet-based aftercare programme and motivate them to follow through with it. It is therefore self-explanatory for participants, as specifically devised video clips illustrate the rationale, the requirements for participants and the course of the aftercare programme. In order to stimulate emotional learning, patients are simulated by actors. Similar means of patient information and preparation have been tested previously by our group [26]. Web application and videos were developed in cooperation with the Knowledge Media Institute of the University of Koblenz-Landau and the Media Centre of the Johannes Gutenberg University Mainz.

The theoretical background of the internet-based aftercare programme is modelled after supportive expressive therapy (SET) by Luborsky [24]. The central part of SET is the ‘Core Conflictual Relationship Theme (CCRT)’ identifying recurrent and maladaptive patterns of relationships. The Identification of maladaptive interpersonal episodes with colleagues and supervisors helps to understand conflicts at the workplace and to develop potential solutions. As writing about significant own emotional experience improves the physical and mental health [27], participants are instructed to write about current (or past) meaningful interpersonal interactions with a focus on the workplace once a week, over a 12 weeks period.

Participants choose a certain day, on which they get a writing instruction (‘writing-impulse’) from their online-therapist, a trained psychologist. The writing-impulses should stimulate the participants to write their text as a diary on the web application. Participants may write one diary entry per week, and the entries are exclusively shared between the participant and the online-therapist. Participant and online-therapist communicate only via the internet-platform. Usually the participant receives an individual feedback from the online-therapist, within 24 h. All writing-impulses are personalised, depending on the last diary entry. Only the first one is standardised for all participants asking participants to describe three social encounters especially related to work. Participants are asked to note their wish in the situation, the reaction of the others and the reaction of the self. In the following weeks participant and online-therapist work out the potentially maladaptive pattern of relationship. The online-therapist also provides encouragement and support. At all times the participants and online-therapists have the opportunity to consult all instructions and diary entries. After the end of the online- aftercare participants may save the conversation as a PDF file (‘Take-home therapy-book’). The three online-therapists are psychologists/ psychotherapists in training, respectively certified in psychodynamic therapy and obtain supervision by a senior psychotherapist experienced in SET.

### Assessments

In order to compare study participants with non-participants, routine data are collected of all inpatients, who are admitted to the rehabilitation clinic during the period of recruitment (T0). These include demographic data (e. g. age, sex), diagnosis and work ability resp. level of functioning at discharge.

In further assessments, only participants of the study are included after they have given written consent. These questionnaires are given online with SoSci Survey [28] at www.soscisurvey.de and all data are assessed pseudonymous. Assessments will be conducted after discharge of inpatient rehabilitation, before the start of the online aftercare (baseline = T1), 1 week after the last writing-impulse is given (post-intervention = T14) as well as during the 12 weeks of the intervention (T2 to T13).

### T1 and T14 assessments

A subscale of the Patient Health Questionnaire (PHQ-9) [29] measures depression, the General Anxiety Disorder Scale (GAD-7) [30] assesses generalized anxiety. The Subjective Prognosis of Work Ability Scale (SPE) measures subjective prospects of work ability [31]. Participants’ ability to work will be measured with the short form of the Work Ability Index (WAI) [32]. By means of the Somatic Symptom Scale-8 (SSS-8) [33] and the EQ-5D [34] the burden of somatic factors and the overall health status will be assessed. Additionally, participants´ psychological and psychosocial stressors are measured by the Perceived Stress Scale (PSS-4) [35] and the corresponding subscale of the Patient Health Questionnaire (PHQ-stress module) [36]. The personal resources will also be assessed with the Oslo Support Scale (OSS-3) [37] and the Brief Resilient Coping Scale (BRCS) [38]. Furthermore the Sheehan-Disability Scale [39] is used to measure the general capability and the Loneliness Scale (LS-S) [40] assesses social integration. In addition to these assessments at T14 participants will be asked about their utilization and their evaluation about helpfulness and overall satisfaction of *‘GSA-Online plus’* (incl. helpfulness of the video clips). Satisfaction will be measured with a modified version (wording adapted for online-intervention) of the ZUF-8 [41]. Furthermore, participants´ willingness to pay for the attendance on *‘GSA-Online plus’* and the costs of the programme will be assessed.

### T2 to T13 assessments

In longitudinal assessments, once a week during the online aftercare (T2-T13), the participants` self-rated work ability is assessed by one item from the Work Ability Index [32] (‘Current work ability compared with the lifetime best.’) and the self-rated health status is measured by one item from the EQ-5D (“Your own health state today.”) [34], both on a Likert-Scale from 0 to 10. In addition, the evaluation of helpfulness and contentedness with the therapeutic feedback will be assessed.

### Objective

In the current trial, we focus on implementing the online aftercare intervention into routine care. Like other follow-up recommendations, referral is done by the medical staff. By adding introductory modules, we enable participants to obtain information about the programme online. The purpose of the implementation study is to examine, how the transdiagnostic aftercare *‘GSA-Online plus’* can be optimally implemented into regular care so that it can be offered to vocationally stressed patients in different areas and clinics of rehabilitation.

The issues are: (1) How many patients receive the aftercare referral of *‘GSA-Online plus’* under conditions of usual care and (2) what is the proportion of patients who follow this referral and use *‘GSA-Online plus’* at least once after inpatient rehabilitation.

Furthermore, we want to determine how different levels of utilization of the aftercare are associated with improvements of the subjective prognosis of work ability and distress. We assume that regular users have a more positive prognosis of work ability and less distress after participation compared to non-regular users, resp. drop-outs. And in an explorative analysis, we want to know how satisfied patients are with the aftercare and how much they would pay for participation.

### Outcomes

#### Primary outcomes

1. Recommendation rate of *‘GSA-Online plus’*. How often will *‘GSA-Online plus’* be recommended as an aftercare after inpatient rehabilitation?
2. Number of patients participating in *‘GSA-Online plus’*. At least 66% of patients with a corresponding recommendation should use *‘GSA-Online plus’* at least once.

### Secondary outcomes

All of the following secondary outcomes are assessed at T1 (study inclusion) and T14 (12 weeks later):

1. Subjective Prognosis of Gainful Employment (SPE)
2. Work ability (WAI)
3. Depression (PHQ-9)
4. Anxiety (GAD-7)
5. Somatic symptoms (SSS-8)
6. Overall health status (EQ-5D-3 L)
7. Psychosocial stressors (PHQ-Stress)
8. Psychological stress (PSS-4)
9. General functioning (Sheehan-Disability Scale)
10. Loneliness (LS-S)
11. Personal resources (OSS-3 and BRCS)

The following secondary outcomes are only assessed at T14 (12 weeks after study inclusion):

1. Overall satisfaction (modified version of ZUF-8) with *‘GSA-Online plus’* as well as helpfulness and utilization
2. Evaluation and utilization of the video clips
3. Willingness to pay and amount of payment for *‘GSA-Online plus’*

A weekly regular monitoring takes place at T2 till T13 (12 weeks during intervention):

1. Self-rated health status (Item drawn from the EQ-5D)
2. Self-rated work ability (Item drawn from the WAI)
3. Satisfaction and helpfulness of the therapeutic feedback

### Sample size

Utilization of *‘GSA-Online plus’* will be examined under conditions of usual care. We will assess how often *‘GSA-Online plus’* will be recommended as an aftercare after inpatient rehabilitation compared to normal aftercare programmes. Therefore, no sample size will be defined. During the period of recruitment all rehabilitants will be included, who obtain an aftercare recommendation of *‘GSA Online plus’* and have the intention to participate. On the basis of the official statistics of the number of treated rehabilitants in the participating clinics and a participation rate of 66% in our prior study we anticipate *N* = 212 participants in all three clinics over a period of 9 months.

### Statistical analysis

Frequencies and descriptive statistics will be used to evaluate recommendation rate and utilization of *‘GSA-Online plus’*. The differential efficiency of subjective prognosis of gainful employment and depression will be analysed with repeated measures ANOVA and regression analysis. Willingness to pay and the utilization criteria of online aftercare will be reported with frequencies and descriptive statistics.

## Discussion

While internet-based interventions have been demonstrated to be efficacious, drawbacks refer to participation and implementation into clinical practice. In clinical practice, participation rates and compliance have been found to be less than satisfactory [42]. Thus, most programmes have not found a place in usual care. Dissemination and implementation of efficacious, evidence-based treatments into medical practice has become a growing issue for mental health care [43]. Therefore it is important to investigate how interventions evaluated in RCTs with selected participants, usually recruited over the internet, can be transferred into routine clinical care, and whether the health care system benefits from such developments. Thus, we implemented a transdiagnostic programme found efficacious in a previous RCT [25] into clinical practice. However, in order to be successfully implemented in routine clinical care, an online intervention needs to be self-explanatory, i.e. easy to handle by busy and changing staff members. Therefore, we augmented the programme by additional exploratory and motivational videos on the study web application to familiarize potential participants and medical professionals with the internet-based aftercare programme. Based on our previous experience with a considerable number of drop-outs from the programme we also took care to motivate them to follow through with it [26]. As other aftercare interventions, referrals to *‘GSA-Online plus’* are made by the physicians in charge. The focus of evaluation is on acceptance of the programme, both by the physicians in charge and the patients. The lack of a control group may be seen as a limitation. However, we take care to specify who gets referred, respectively accepts the recommendation compared to those who do not get the recommendation, respectively refuse to participate. Additionally, we assess the frequency and subjective evaluation of participation in order to model treatment gains based on regular participation. If our implementation study proves to be successful, a next step will be to resolve legal requirements and financial resources necessary to implement *‘GSA-Online plus’* as a regular aftercare programme for vocationally stressed inpatients of medical rehabilitation.

## Abbreviations

BRCS: Brief Resilient Coping Scale
CCRT: Core Conflict Relationship Theme
EQ-5D-3 L: EuroQol
GAD-7: General Anxiety Disorder Scale
GSA-Online plus: Gesund und Stressfrei am Arbeitsplatz [Healthy and Without Stress at the Workplace]- Online plus
GSA-Online: Gesundheitstraining Stressbewältigung am Arbeitsplatz [Health Training Stress Management at the Workplace]- Online
LS-S: Loneliness Scale
OSS-3: Oslo Support Scale
PHQ: Patient Health Questionnaire
PSS-4: Perceived Stress Scale (short-form)
RCT: Randomised controled trial
SET: Supportive expressive therapy
SPE: Subjective Prognosis of Work Ability Scale
SSS-8: Somatic Symptom Scale
WAI: Work Ability Index
ZUF-8: Fragebogen zur Messung der Patientenzufriedenheit [CSQ-8: Client Satisfaction Questionnaire]
